# The consumption of unhealthy foods by Brazilian children is influenced by their mother’s educational level

**DOI:** 10.1186/1475-2891-13-33

**Published:** 2014-04-03

**Authors:** Silvia Regina Dias Medici Saldiva, Sonia Isoyama Venancio, Andréia Cardoso de Santana, Ana Lucia da Silva Castro, Maria Mercedes Loureiro Escuder, Elsa Regina Justo Giugliani

**Affiliations:** 1 Instituto de Saúde, Secretaria do Estado da Saúde de São Paulo, Rua Santo Antônio, 590, Bela Vista, 01314-000 São Paulo, SP, Brazil; 2 Departamento de Pediatria e Puericultura, Universidade Federal do Rio Grande do Sul, Faculdade de Medicina, Rua Ramiro Barcelos, 2400, Santa Cecília, 90035-003 Porto Alegre, RS, Brazil

**Keywords:** Supplementary feeding, Food habits, Infant, Cross-sectional study

## Abstract

**Objective:**

To evaluate the association between the consumption of unhealthy foods in children under one year and the education level of the mothers, data obtained from participants of the II Survey on the prevalence of breastfeeding in the Brazilian capitals and the Federal District in 2008 was analyzed.

**Methods:**

During the second stage of the campaign for multi-vaccination, a questionnaire on food consumption in the last 24 hours was given to mothers or guardians of children under one year old. We analyzed the consumption of unhealthy foods according to age group, maternal education, region of residence and breastfeeding status. The state capitals and the Federal District were grouped according to the five macro-regions of the country (North, Northeast, Southeast, South and West). Processed juice, soda, coffee, cookies/salted snacks and sugar and/or honey were defined as unhealthy foods. Prevalence ratios (RP) for the association between the consumption of unhealthy foods and maternal education were estimated using Poisson regression models. Results: The study included 34,366 children. The consumption of sweet foods started early and was predominant until the age of six months; after this age, the consumption of biscuits and/or snacks became more prevalent. The consumption of these foods also differs in relation to the macro-region of residence. Consumption of unhealthy foods was higher among mothers with lower education levels.

**Conclusions:**

The consumption of unhealthy foods by Brazilian children under one year old was high, indicating a need for developing effective strategies to combat the consumption of unhealthy foods in Brazilian children as a way of preventing obesity and other future disorders.

## Introduction

In recent decades, a significant increase in the prevalence of obesity has occurred worldwide mainly in developing countries [1]. Approximately 43 million children under 5 years old are overweight or obese, and 80% of these children are living in developing countries [2]. In Brazil, data from the National Demographic and Health Survey in 2006 indicated that 7.3% of children under 5 years old are overweight [3]. Data from the Household Budget Survey (POF/2009) [4] showed that one in three children between the ages of 5 and 9 years old were overweight according to the World Health Organization guidelines [5]. The development of obesity during infancy and early childhood is associated with rapid infant weight gain, infant-feeding practices, sleep duration, the child’s diet, physical activity, sedentary practices and racial/ethnic differences [6]–[8]. Despite the inability of the authors to demonstrate a clear association between the age when solid foods are introduced and obesity, the conclusion of a systematic review examining the association between the timing of solid food introduction and obesity during infancy in developed countries recommended that a whole family approach to obesity prevention is the most effective [9].

In this context, it is essential to promote healthy eating habits early in life, which includes exclusive breastfeeding (EBF) during the first six months of life and, after this age, the introduction of appropriate complementary feeding (WHO,2003) [10]. EBF is relatively uncommon worldwide; only 36% of infants from 46 developing countries are exclusively breastfed, less than one-third of children between the ages of 6 and 23 months meet the minimum dietary diversity, and only 50% of children receive the minimum number of meals [10]. Data from the study of African-American infants and young children participating in the US Infant Care and Risk of Obesity Study show that the rates of inappropriate feeding age are high and are associated with a higher daily energy intake and an increased odd of high infant weight-for-length [11]. A systematic review examining the types of foods introduced during complementary feeding and the risk of childhood obesity showed that higher energy intake during complementary feeding was associated with a higher Body Mass Index (BMI) in childhood [12].

In Brazil, despite advances, data from the Second National Survey on the Prevalence of Breastfeeding (PPMA II), which was conducted in all Brazilian capitals and the Federal District in 2008, showed that only 41% of children ages zero to six months were exclusively breastfed. Regarding complementary feeding, the survey found that 21% of children between the ages of 3 and 6 months experienced an early introduction of foods, and, even more alarming was the finding that these children consumed a large amount of unhealthy foods such as cookies, snack foods and soft drinks during their first year of life (MS, 2009) [13].

There is some evidence concerning the association between parental education and the consumption of unhealthy foods. The IDEFICS cohort study in Europe analyzed data from children aged 2 to 9 years old and showed that low parental education level was associated with higher intake of sugar-rich and fatty foods among children, while high parental education levels were associated with higher intake of low-sugar and low-fat foods [14]. However, the studies conducted in less affluent parts of the world focused primarily on the risk of undernutrition, and very few studies addressed the determinants of obesity [15]. Moreover, most of the studies conducted in this field focus on preschool and school age groups, indicating that factors in early childhood (< one year) could be important for understanding the determinants of obesity in later life and provide insights into the importance of adequate nutrition during early life in obesity prevention.

Considering the heterogeneity of the educational level in Brazil and the availability of data from the large survey conducted in PPMA II, we designed the present study to evaluate the association between the mother’s education level and the consumption of unhealthy foods among children younger than one year old.

## Methods

For this study, we utilized the data contained in the database of the Ministry of Health on the PPMA II, which was conducted in all state capitals and the Federal District during the second stage of the multi-vaccination campaign in 2008 [13]. Survey samples were obtained in clusters with two-stage draw with a probability proportional to the size of the group. The sampling plan was based on information from the 2007 vaccination campaign, which was provided by the Municipal Health Department of the capitals and the Federal District, and a survey conducted in 1999 provided information regarding the prevalence of EBF in these places [16]. First, the vaccination posts in each capital were selected, and then, the number of children in each cluster (post) was selected.

The data collection instrument included closed questions about the dietary intake of the children. The interviews had to be conducted quickly so as not to interfere with the vaccination campaign. Therefore, most questions pertaining to feeding were dichotomous (yes/no) Questions about breastfeeding and consumption of liquids other than human milk (water, tea, milk powder, milk box, fruit juice) were collected to characterize breastfeeding practices; the types of food consumed (porridge, mash, soup, homemade food, fruits, vegetables, meat, beans) were included to analyze the introduction of complementary foods and some unhealthy foods, commonly consumed in Brazil and not recommended by the Ministry of Health’s guide for infant feeding under 2 years (soda, processed juice, coffee, cookies/salted snacks, foods sweetened with sugar or honey) were introduced as markers. The instrument follows the WHO recommendations for obtaining indicators on infant feeding practices in population studies, such as using current data (current status) via 24 h recall that aims to minimize possible recall bias [17]. Characteristics of the children and mothers were collected to examine associations between personal characteristics and feeding practices. Trained interviewers administered the questionnaires to all mothers or caregivers of children less than 12 months of age. Those responsible for research in the municipalities typed the data into an application online. The database was exported and analyzed using Stata 10.1 Stata Corp LP, Texas USA and the charts and tables were prepared using Microsoft Office Excel 2003.

Specific procedures for data analysis from complex probability-sample surveys were used to obtain descriptive statistics. We analyzed food intake according to: the age of the child (0–6 and 6–12 months); maternal education: college (≥13 years of study), high school (between 10 to 12 years of study), elementary school (up to 9 years of study) and no schooling; the region of residence (North, Northeast, Southeast, South and Center-West); and breastfeeding status. Next, we estimated the prevalence ratios (PR) for the association between the consumption of each unhealthy food and maternal education, using unhealthy food consumption as a “proxy” for socioeconomic status, which would directly influence care during childhood [18]. The significance of the associations between unhealthy food consumption and the information obtained by the questionnaire was verified by conducting a Poisson regression with robust variance for complex samples, as recommended for cross-sectional data with non-rare outcomes [19].

We considered the indicators of unhealthy food consumption in the day before the interview the dependent variable. The following foods were classified as unhealthy: processed juices, soda, coffee, cookies and/or salted snacks and sugar and/or honey. The main independent variable was maternal education (college education, high school, elementary school and no education); additional explanatory variables were also considered, such as: the “age of the child” in days; “maternal work” (work outside the home, no work/maternity license); “maternal age” (<20 years, 20–34 years, ≥35 years); “breastfeeding” (yes, no); region of residence (North, Northeast, Midwest, Southeast, South). The independent effect of explanatory variables with a significance level of ≤20% on chi-square testing was adjusted in the multiple regression models.

The Ethics Committee of the Institute of Health approved this study’s protocol (protocol 001/2008 of 06/05/2008) after consulting with the National Commission for Ethics in Research (CONEP).

## Results

This study included 34,366 children, and their characteristics are presented in Table 1. The majority of the interviews were conducted with mothers (88%), whereas fathers were interviewed in 7.6% of the cases. In the remaining cases, the relative responsible for the child completed the interview.

**Table 1:** Characteristics of mothers and children under 1 year of age in the Brazilian capitals and the Federal District, 2008

**Sample characteristics**	**N**	**%**
**Child’ sex**		
Female	17,038	49.6
Male	17,328	50.4
**Low birth weight**		
Yes	2,695	8.3
No	29,915	97.7
**Breastfeeding**		
Yes	26,715	78.4
No	7,370	21.6
**Child’ age (months)**		
0 to 5.99	18,494	54.8
6 to 11. 99	15270	45.2
**Maternal age**		
<20 years	5,060	14.7
20 to 35 years	21,434	62.4
≥35 years	3,153	9.2
No information	4,719	13.7
**Maternal work**		
Outside the home	5,757	16.7
Not working/Maternity license	22,496	65.5
No information	6,113	17.8
**Maternal education level**		
No schooling	644	1.9
Elementary school	10,851	31.6
High school	14,062	40.9
College	4,284	12.5
No information	4,525	13.2
**Region of residence**		
North	7,951	23.1
Northeast	12,139	35.3
Midwest	4,597	13.4
Southeast	6,405	18.6
South	3,274	9.5

Table 2 shows the frequency of unhealthy food consumption by age, region of residence and breastfeeding status. We verified that the consumption of sweetened foods began early and was predominant during the first six months of child’s life; after this age, the consumption of cookies and/or salted snacks was more prevalent. Over 70% of children between the ages of 9 and 12 months consumed cookies and/or salted snacks on the day before the interview.

**Table 2:** The frequency of unhealthy food consumption by age group, breastfeeding status and region of residence in children under 1 year of age in the Brazilian capitals and the Federal District, 2008

	**Processed juice**		**Sodas**		**Coffee**		**Cookie/Salted Snacks**		**Sugar/Honey**	
	**n**	**%**	**n**	**%**	**n**	**%**	**n**	**%**	**n**	**%**
**Age group**	*p = 0.0001*		*p = 0.0006*		*p = 0.0001*		*p = 0.0001*		*p = 0.0001*	
0 to 5.9 m	289	1.6	70	0.8	96	0.5	867	4.7	2638	14.3
6 to 12 m	1797	11.8	1261	8.2	1040	6.8	8981	59	7609	49.9
**Breastfeeding status**	*p = 0.0001*		*p = 0.0001*		*p = 0.0001*		*p = 0.0001*		*p = 0.0001*	
Yes	1341	5.1	910	3.5	769	2.9	6343	24.3	6498	24.8
No	727	10.0	405	5.6	359	4.9	3432	47.4	3665	50.4
**Child’ sex**	*p = 0.9044*		*p = 0.8937*		*p = 0.2215*		*p = 0.0498*		*p = 0.3043*	
Female	1030	6.1	654	3.9	543	3.2	5001	29.9	4961	29.7
Male	1056	6.2	677	4.0	593	3.5	4847	28.6	5286	31.1
**Mothers’ age**	*p = 0.7193*		*p = 0.0008*		*p = 0.0034*		*p = 0.7381*		*p = 0.0165*	
<20 years	341	6.8	259	5.2	228	4.6	1408	28.3	1571	31.5
20 to 35 years	1233	5.8	775	3.7	660	3.1	5934	28.2	6103	28.9
≥35 years	138	4.4	87	2.79	68	2.2	821	26.5	809	26.0
**Maternal work**	p = 0.0237		*p = 0.0001*		*p = 0.0001*		*p = 0.0001*		*p = 0.0001*	
Outside the home	428	7.5	228	4.0	193	3.4	2095	37.0	2020	35.6
Not working/Maternity license	1237	5.6	854	3.8	732	3.3	5770	26.0	6057	27.3
**Maternal education level**	*p = 0.0003*		*p = 0.0001*		*p = 0.0001*		*p = 0.0001*		*p = 0.0001*	
No schooling	65	10.4	38	6.0	39	6.2	215	34.3	245	38.9
Elementary school	702	6.5	539	5.0	483	4.5	3218	30.2	3460	32.4
High school	769	5.5	459	3.3	385	2.8	3794	27.5	3950	28.5
College	208	4.9	96	2.3	64	1.5	1028	24.4	934	22.1
**Region of residence**	*p = 0.0001*		*p = 0.2944*		*p = 0.061*		*p = 0.0001*		*p = 0.0001*	
North	633	8.0	485	6.1	387	4.9	1904	24.3	2444	31.0
Northeast	409	3.4	229	1.9	276	2.3	3432	29.0	4052	34.2
Midwest	369	8.2	200	4.4	156	3.5	1422	31.7	1177	26.2
Southeast	421	6.7	275	4.3	176	2.8	2022	32.2	1686	26.7
South	254	7.9	142	4.4	141	4.4	1068	33.3	888	27.7

The consumption of unhealthy foods also differs between regions of residence. The consumption of processed juice, soda and coffee occurred less frequently in the Northeast. Additionally, a higher consumption of cookies and/or salted snacks was observed in the South, and a higher consumption of sweetened foods was observed in the Northeast. It was also found that breastfed children under one year old received unhealthy foods less frequently compared to children who were not breastfed (Table 2).

It is important to notice that a greater consumption of unhealthy foods does not necessarily imply that other types of foods were not being given to the children. Vegetables and fruit consumption was influenced by maternal education as well. Mothers with college degrees gave their children more fruits and vegetables than mothers with only high school or elementary school educations *(p = 0.001).* Interestingly, the differences between the highest and the lowest education levels were not significant for healthy foods, which was in contrast with the findings obtained for unhealthy foods (data not showed).

**Figure 1:**
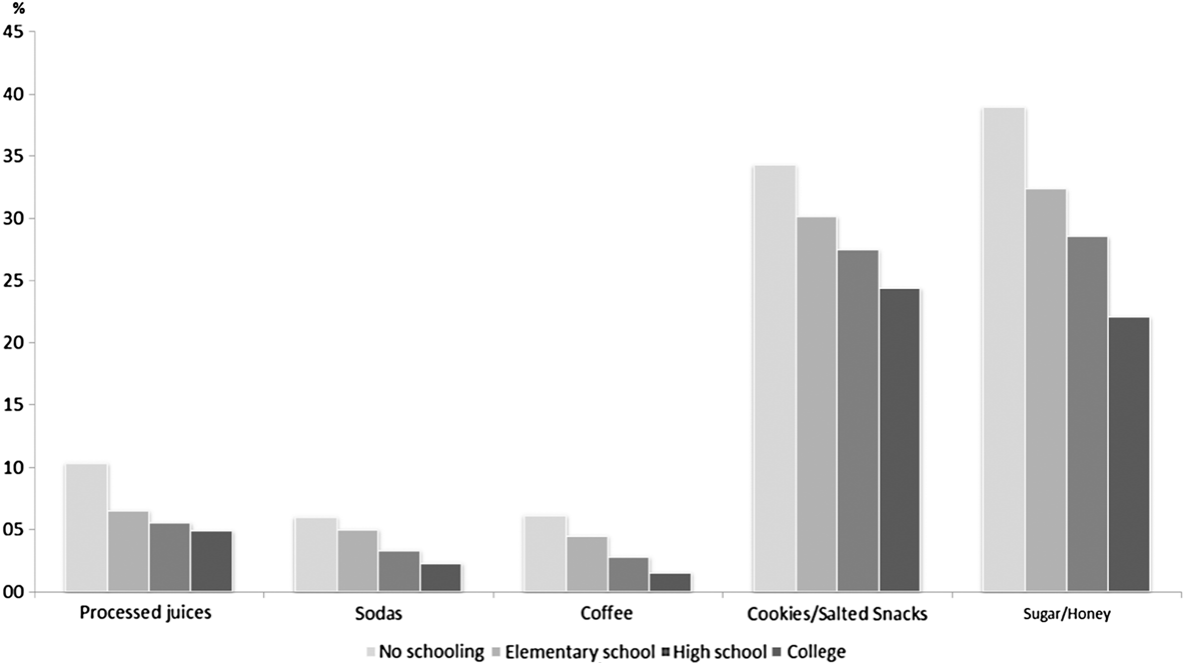
The frequency of unhealthy food consumption according to the mother’s educational level in children between the ages of 0 and 12 months in the Brazilian capitals and the Federal District, 2008.

Figure 1 shows the frequency of unhealthy food consumption by children under 1 year old according to maternal education, which indicates that the higher the mother’s education level the lower the consumption of unhealthy foods. Children of mothers with no formal education consumed twice as much processed juice, soft drinks and coffee compared to the children of mothers with college degrees.

Table 3 shows the crude and adjusted analyzes of the association between unhealthy food consumption and maternal education. The consumption of processed juice, coffee and sugar and/or honey was negatively associated with the educational level of the mother. Children of uneducated women consumed between 2 and 3 times more of these foods more frequently when compared with children of women with higher education levels. The consumption of soft drinks and cookies and/or salted snacks was highest among children of women with only an elementary education. It is important to notice that the rates of unhealthy food consumption across the different education levels were not markedly different after adjusting for other possible confounders. These findings indicate that the association between unhealthy feeding and a mother’s lower education level is robust.

**Table 3:** **Estimates of the prevalence ratio, tendency *(p) *** **and confidence intervals (CI95%) by crude and fitted analyses for the association between the unhealthy food consumption and maternal education level, 2008**

**Food**	**Crude**		**Fitted**	
	**PR**	**CI**	**PR**	**CI**
**Processed juice *(p = 0.0008)***				
College	1	-	1	-
High school	0.87	0.68 - 1.1	1.02	0.79 - 1.31
Elementary school	1.15	0.91 - 1.46	1.36	1.07 - 1.74
No schooling	1.56	0.98 - 2.50	1.99	1.12 - 3.54
**Sodas *(p = 0.0001)***				
College	1	-	1	-
High school	1.44	1.03 - 2.01	1.73	1.22- 2.47
Elementary school	2.51	1.81 - 3.50	2.83	1.99 - 4.03
No schooling	2.30	1.26 - 4.21	2.34	1.10- 4.99
**Coffee *(p = 0.0001)***				
College	1	-	1	-
High school	1.96	1.32 - 2.92	2.10	1.34 - 3.28
Elementary school	3.06	2.07 - 4.51	3.10	1.99 - 4.84
No schooling	4.20	2.19 - 8.06	3.23	1.41 - 7.42
**Cookies/Salted Snacks (*p = 0.0001)***				
College	1	-	1	-
High school	1.10	1.01 - 1.21	1.30	1.20 - 1.41
Elementary school	1.23	1.12 - 1.34	1.41	1.30 - 1.53
No schooling	1.36	1.11 - 1.66	1.27	1.04 - 1.54
**Sugar or honey *(p = 0.0001)***				
College	1	-	1	-
High school	1.24	1.13- 1.36	1.40	1.28 - 1.54
Elementary school	1.44	1.31 - 1.58	1.61	1.46 - 1.78
No schooling	1.72	1.41 - 2.07	1.9	1.55 - 2.34

Variables controlled for: child’ sex, maternal age, maternal work, breastfeeding status, region of residence and age of the child in days.

## Discussion

The results of this study showed that unhealthy foods are present in high frequency in the diets of children under one year old and that this behavior is associated with low maternal education. These results are consistent with the findings of other studies on infant feeding conducted in Brazil as well as those of studies in other countries [20,21].

The presence of unhealthy foods in the diets of children was analyzed in three studies in the state of São Paulo. A survey conducted in 136 counties detected that 63% of children between 6 and 12 months consumed porridge, which, in most cases, has sugar added during the preparation [22]. In the city of São Paulo, a study revealed that the introduction of food occurs before 6 months of age especially sweets, and that 69% of children consumed soft drinks at 12 months of age [23]. Additionally, in the city of São Paulo, a study on a random sample of children from birth to 59 months showed that family income influenced the consumption of processed foods, and the children from families with lower incomes were more likely to consume sugar [24].

The consumption of unhealthy foods has also been demonstrated in children under five that are beneficiaries of Bolsa Família (Brazilian program of cash transfers for families in poverty and extreme poverty) in the Brazilian semiarid region. These children were three times more likely to consume sweets than children not receiving this benefit, which suggests that income and maternal education may have an important influence on food choice [25].

Weaning represents an opportunity to promote lasting dietary habits. An optimal transition from milk to healthy table and family foods will help to establish healthy eating habits that may affect food preferences into adulthood [26]. Therefore, this period may be considered one of the determinants of healthy eating habits, which, ultimately, may prevent diet-related diseases [27].

In countries such as The United States, infants and young children obtain most of their nutrients from infant formulas and/or fortified cereals and other fortified foods. While these foods provide a substantial proportion of a child’s micronutrient requirement, there are some indications that excessive intake of these types of foods should be avoided [28]. Overweight and obese children are a public health problem, and diets that exceed dietary guidelines for fat, cholesterol, added sugar, saturated fatty acids and sodium and are low in fiber must be avoided to prevent diseases [29]. The consumption of sugar is associated with dental caries [30] and a higher risk of developing childhood obesity [31,32]. In addition, low quality diets may cause micronutrient deficiencies due to their lower nutrient content when compared to products lower in sugar [33,34]. Excessive salt intake during childhood increases the risk for cardiovascular disease in adulthood. High sodium intake during the first 6 months of life has been associated with higher blood pressure [35-37]. Therefore, excessive exposure to early obesogenic diets may also influence appetite regulation and the ability of the hypothalamic neural circuit to regulate appetite by inducing permanent changes in the complex pathways that link the hypothalamus, gastrointestinal tract and adipose tissue [38]. These findings reinforce the importance of promoting healthy nutritional habits during childhood.

In Brazil, the prevalence of breastfeeding and exclusive breastfeeding, though still below the levels recommended by the WHO, has been gradually increasing in recent decades, as a result of major advances by the National Program to Encourage Breastfeeding (PNIAM) to promote breastfeeding since it was founded in 1981 [39,40]. However, public strategies to promote healthy complementary feeding were adopted much more recently [41]. Examples of these strategies are in the publication of “Dietary Guidelines for Children Under Two Years - Ten Steps to Healthy Eating” in 2002 (revised in 2010) [42] and the formulation of the National Strategy for Healthy Complementary Feeding (ENPACS) [43], which aim to support and promote healthy infant feeding at the primary health care level.

This study confirmed that children under one year consume high amounts of ultra-processed foods such as biscuits and soft drinks. It is important to know the deleterious effects of these foods. Because they are formulated to be durable, affordable and easy to eat, they contain excessive amounts of oils, fats, flour, starch, sugar and salt, as well as preservatives, stabilizers, flavorings and colorings. They feature high energy density, low nutritional value and scarcity of fiber, which are all characteristics that increase the risk for obesity, diabetes and cardiovascular disease [44,45].

An association between unhealthy food consumption in children and socioeconomic status of their families was also found in developed countries. In Bristol, UK, an inverse relationship between maternal education and the consumption of unhealthy foods, such as sausage and burgers, by 10-year-old children was found; and, in contrast, there was a positive relationship between the consumption of fruits and vegetables and maternal education [46]. In California, United States of America, a study conducted among parents of children who attended a school for low-income families showed that the most common barriers to healthy food consumption were their high cost and the ease of access to fast food. Furthermore, it was shown that many parent’s basic knowledge about nutrition came primarily from television, radio, magazines and newspapers, demonstrated that they were easily influenced by the media [47].

In a population-based cohort study with Brazilian children, it was found that maternal education has an effect on a child’s health, which is partly independent from that of other socioeconomic factors; it was also suggested that maternal care is more important than the biological characteristics of the mothers, because stronger effects were observed for a child’s health outcome later (post neonatal mortality, hospital admissions and nutritional status) rather than earlier (birth weight, perinatal mortality) [48].

The high consumption of unhealthy foods in children less than one year old is most likely a reflection of changes in food expenditures in the Brazilian population, which was identified by the Household Budget Surveys (HBS). For example, among the products that showed an increase in the average per capita amount purchased, cola soft drinks occupied a prominent place, which grew by 39.3% between 2002–2003 and 2008–2009 [49]. Another important finding of this survey was that out of the 1,792 kcal available for each person on average, approximately 20% of the kcal come from products considered to be ultra-processed, while 38% of the kcal comes from processed foods [50].

We agree with Victora et al. [48] when they say that the main challenge is how to educate parents to provide healthy nutrition habits for their children, especially in a situation where the parents are undereducated or have deep-seated cultural values. The parent’s attitude towards eating is a determinant of their children’s future diets, and it influences their feeding habits and, consequently, their risk for diet-associated diseases.

One of the major limitations of this study is related to the use of the 24-hour recall with closed questions, which cannot investigate the amount consumed, the frequency of consumption or the introduction of foods into the diet of infants.

This study gave an important contribution to quantify, at a national level, some feeding practices in young children. The results serve as a warning to health professionals, managers and society about the need for developing effective strategies to tackle unhealthy food consumption among Brazilian children as a way to prevent obesity and other health problems in the future. It is hoped that the newly launched strategy “Breastfeeding and Feeding Brazil”, aimed at promoting healthy eating habits early in life at the primary health care level, can help address this important public health problem. Other measures, such as regulating food advertisements, which already occurs with products designed to substitute breast milk [51], has been widely debated by researchers, scientific societies, professional associations, consumer organizations and civil societies in general.

## Conclusion

The results of this paper show that a high consumption of cookies, sugar and processed juices in children less than one year old is associated with maternal education. That is, mothers with lower education levels feed their children more unhealthy foods. We know that to improve complementary feeding, we need to understand the attributes that mothers and caregivers ascribe to foods in their specific cultural setting. Another important aspect to be considered is improving the availability and accessibility of low-cost nutritious complementary foods. In addition, specific policies to regulate the advertising of infant foods need to be adopted because it is known that advertising influences the consumption of unhealthy foods.

## Abbreviations

WHO: World Health Organization; EBF: Exclusive Breastfeeding; PPMA II: Second National Survey on the Prevalence of Breastfeeding; CONEP: National Commission for Ethics in Research; PNIAM: National Program to Encourage Breastfeeding; ENPACS: National Strategy for Healthy Complementary Feeding; HBS: Household Budget Surveys.

## Competing interests

All authors declare that there are no conflicts of interest to disclose.

## Authors’ contributions

SRDM and SIV conceived the paper, conducted the analysis and interpretation of the data, and wrote the manuscript. ACS, ALSC and MMLE contributed to the analysis, and ERJG contributed to the design of the study and writing. All authors revised and approved the final version of the paper for publication.
